# Drawing the neurodegenerative line: plasma biomarkers and retinal OCTs shed light on pathophysiological and diagnostic similarities between Alzheimer's Disease and Multiple Sclerosis

**DOI:** 10.1002/alz.088222

**Published:** 2025-01-09

**Authors:** Dean Zeldich, Chia Hsin Cheng, Yi Guan, Bang‐Bon Koo

**Affiliations:** ^1^ Thomas Jefferson University Hospital, Philadelphia, PA USA; ^2^ Boston University Chobanian & Avedisian School of Medicine, Boston, MA USA

## Abstract

**Background:**

Recent evidence suggests extensive myelin dysfunction in Alzheimer’s Disease (AD), lending to investigation of biomarkers previously implicated in both AD and Multiple Sclerosis (MS) to find objective and obtainable diagnostic screening tools. Glial fibrillary acidic protein (GFAP), Chitinase‐3‐like protein 1 (CHI3L1), Chemokine (C‐X‐C motif) ligand 13 (CXCL13), and neurofilament light chain (Nfl) have been known to mark neuronal pathology in both diseases making them attractive markers. Retinal Optical Coherence Tomography (OCT) becomes a popular diagnostic tool in both conditions as an inexpensive and rapid way of obtaining a window into the cerebrum.

**Method:**

AD (n=3952) and MS (n=1988) patients were selected from the UK‐Biobank using ICD10 codes. Past medical history of strokes and brain cancer were used as exclusionary criteria. Case‐control differences were analyzed in cognitive measures, retinal OCT, plasma proteins, and magnetic resonance imaging (MRI) data. AD subjects were further stratified. Correlation and predictability of plasma markers and retinal OCTs with cognitive and MRI measures were investigated with linear regression models, age‐ and sex‐controlled.

**Result:**

Stable AD, MS, and AD converters showed significantly worse cognitive performances compared to controls. GFAP and Nfl were significantly elevated in MS (additionally, CHI3L1), AD patients, and AD converters as early as 5 to 10 years before diagnosis. Retinal OCT markers such as retinal nerve fiber thickness (RNFT) were significantly decreased in MS patients, while less clear patterns were observed for AD and converters. MRI measures obtained 10 years after baseline visit revealed significantly smaller hippocampal volumes and more white matter hyperintensities in both AD and MS patients compared to controls. Overall, retinal OCT measures (i.e., thinner RNFT, smaller optic disc diameter) significantly correlated with worse cognitive outcomes and elevated protein markers for both groups. While certain measures of cognitive decline (e.g., mean reaction time, numeric memory) better correlated with well‐established MRI hallmarks for neurodegeneration.

**Conclusion:**

Inexpensive and non‐invasive biomarkers such as plasma proteins and retinal OCT measures carry significant prognostic potential in AD and MS while allowing for a deeper characterization of the neuronal and glial pathology in both diseases.

